# Epiglottitis Caused by Pasteurella multocida: An Uncommon Presentation

**DOI:** 10.7759/cureus.81365

**Published:** 2025-03-28

**Authors:** Manogna Pendyala, Purnoor Kaur, Mansi Jain, Ayesha Tahir, Vinod Khatri

**Affiliations:** 1 Department of Internal Medicine, Mercy Health - St. Vincent Medical Center, Toledo, USA; 2 Department of Pulmonary and Critical Care Medicine, Mercy Health - St. Vincent Medical Center, Toledo, USA

**Keywords:** dysphagia, edematous arytenoids, epiglottitis, intubation, steroids

## Abstract

*Pasteurella multocida* is commonly associated with skin and soft tissue infections after animal bites and is rarely associated with epiglottitis, which is a life-threatening condition. Here, we describe a patient with epiglottitis secondary to *Pasteurella* infection and bacteremia who required transnasal fiberoptic intubation and was treated with intravenous (IV) ampicillin-sulbactam, who responded well to the treatment with complete resolution of symptoms.

## Introduction

*Pasteurella* are gram-negative coccobacillus that are the primary commensals of animals, mostly dogs and cats [1]. It can be transmitted to humans through bites or scratches, via the respiratory tract from infectious droplets [2]. They can cause soft tissue infections, including cellulitis, which can progress to serious necrotizing infections [2,3]. The mechanisms of the soft tissue infections involve a bacterium polysaccharide capsule that resists phagocytosis, adhesion, and invasion of the host tissue and production of *Pasteurella multocida* toxin (PMT), which contributes to tissue damage and inflammation [4]. While soft tissue infections are the most common manifestation, it has also been documented as a rare cause of epiglottitis [5], and there are only a few case reports published as per our knowledge. We present a case report of epiglottitis due to *Pasteurella *with rapid progression and airway deterioration requiring intubation.

## Case presentation

A 68-year-old Caucasian male with a known history of prior tobacco use, essential hypertension, paroxysmal atrial fibrillation, and type 2 diabetes mellitus presented to the emergency department with complaints of hoarseness and progressive dysphagia over one day. He initially experienced difficulty swallowing his pills in the morning, which rapidly worsened throughout the day, accompanied by significant swelling in his oral cavity and throat. His current medications include Eliquis, amiodarone, lisinopril, metoprolol, atorvastatin, metformin, and omeprazole, with no documented food or drug allergies.

**Figure 1:**
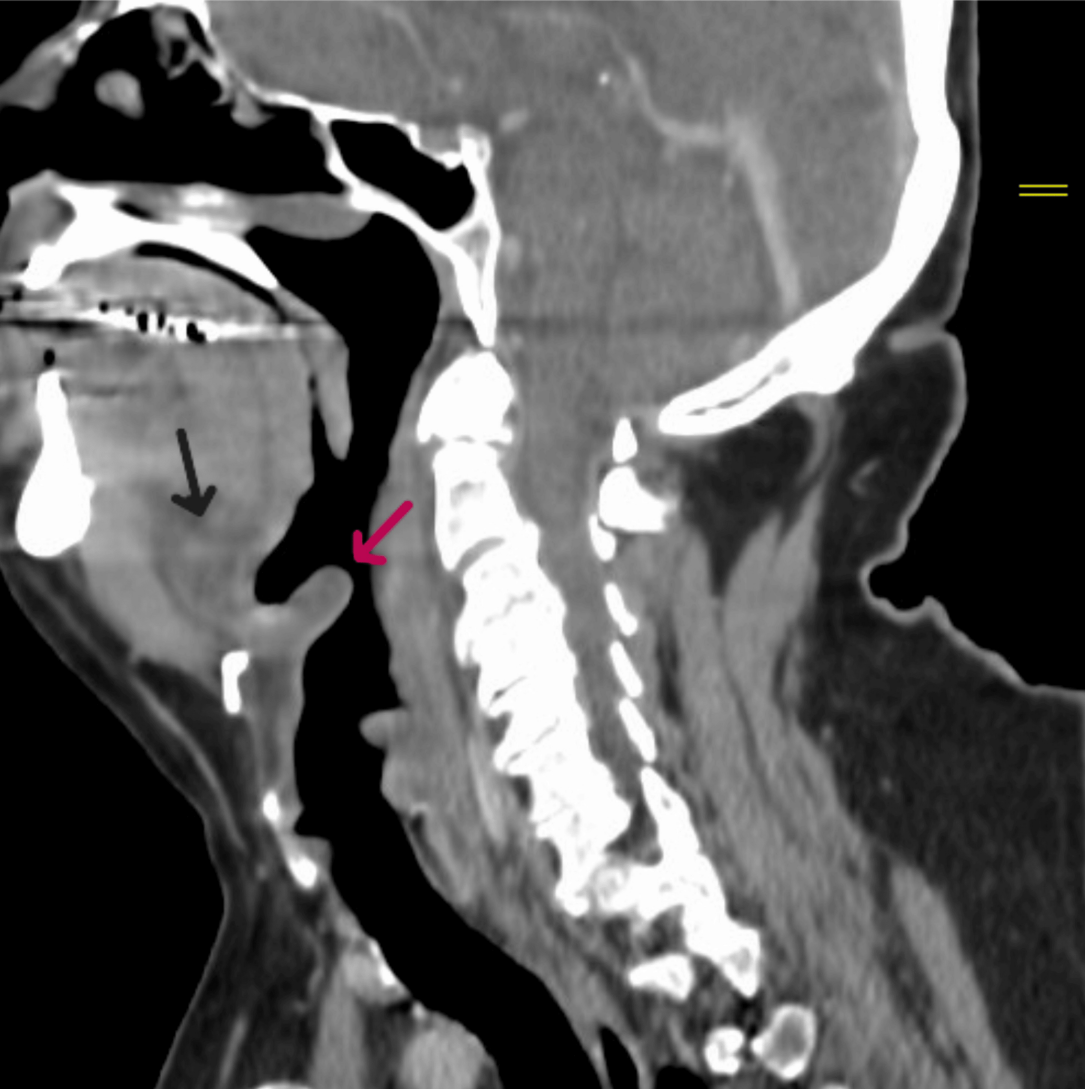
CT image showing edematous epiglottis (red arrow) along with edema of the floor of the mouth (black arrow) CT: computed tomography

On initial evaluation in the emergency room, the patient reported pain and swelling in his mouth and throat but was otherwise tolerating oral secretions and maintaining his airway. He was afebrile, hemodynamically stable, and not in respiratory distress. Physical examination revealed significant edema of the floor of the mouth and sublingual tissues, along with elevation of the tongue, making visualization of the posterior pharynx difficult. He was unable to close his mouth completely. Laboratory tests showed leukocytosis (white blood cell count: 16.8 k/uL, normal range: 3.5-11.3 k/uL) and elevated C-reactive protein (CRP) (148 mg/dL, normal range: 0.0-5.0 mg/dL). A contrast-enhanced computed tomography (CT) scan of the soft tissues of the neck demonstrated an edematous epiglottis with mucosal edema along the left aryepiglottic fold and left lateral oropharyngeal mucosa, consistent with acute epiglottitis (Figure 1).

**Figure 2:**
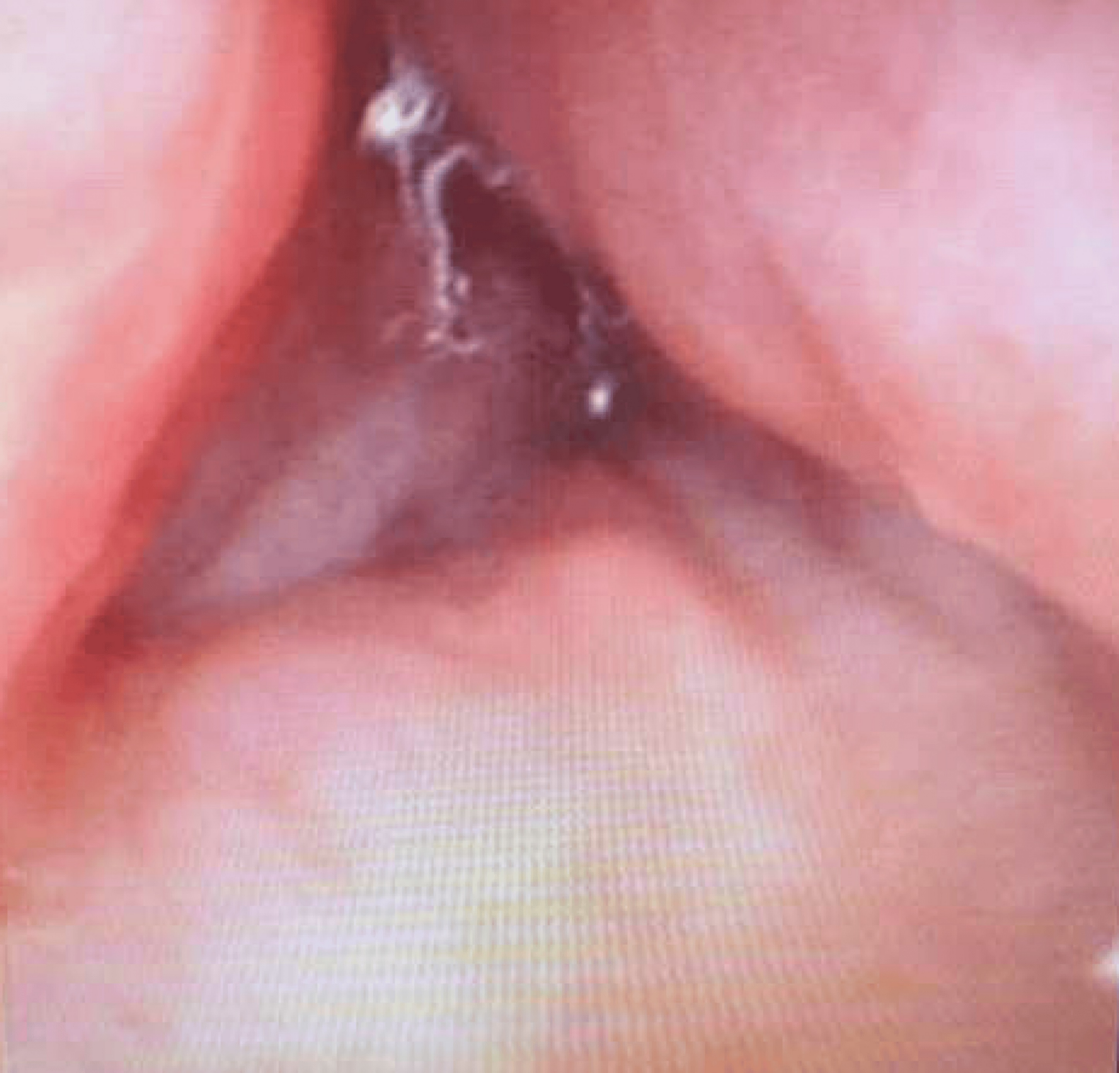
Edematous arytenoids and glottic inlet

**Figure 3:**
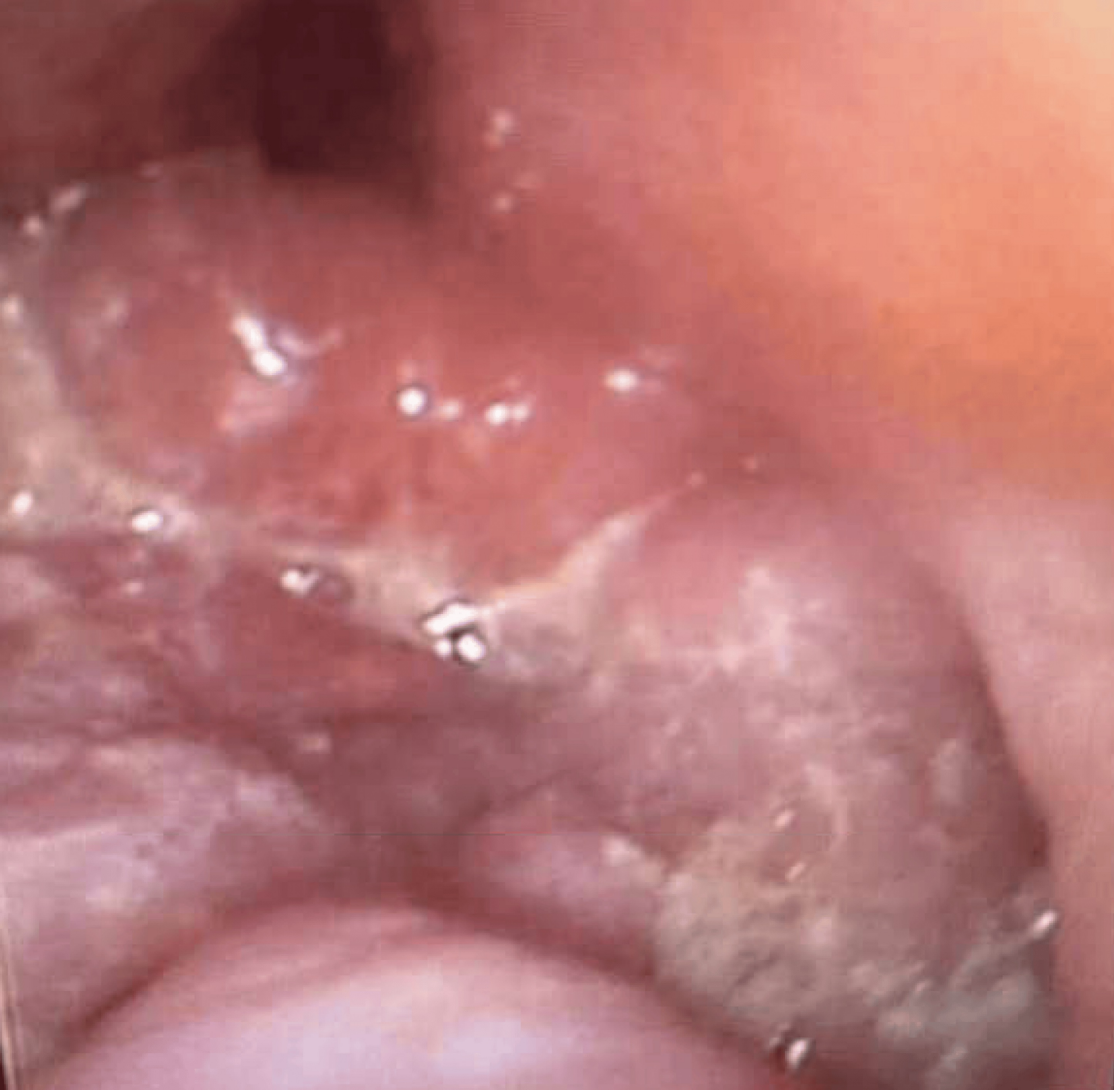
Edematous epiglottis and base of the tongue

The patient was started on intravenous (IV) dexamethasone and empiric antibiotic therapy in the emergency department with vancomycin to cover methicillin-resistant S*taphylococcus aureus *(MRSA) and clindamycin to cover *Streptococcus*, *Staphylococcus*, and *Haemophilus*. Due to worsening dysphagia and edema of the floor of the mouth, with concerns for airway compromise, a difficult airway alert was initiated for intubation. The otolaryngology team performed an awake transnasal flexible fiberoptic intubation in a controlled setting. Fiberoptic examination revealed a posteriorly prolapsing edematous base of the tongue, as well as edema of the epiglottis, arytenoids, and aryepiglottic folds (Figure 2 and Figure 3). The patient was then transferred to the medical intensive care unit (ICU) for continued care.

During his ICU stay, faint, small scratches were noted on his left forearm. He was treated with intravenous steroids and antibiotics, including ceftriaxone and vancomycin. The choice of antibiotics was made due to the better safety profile of ceftriaxone and the risk of *Clostridium difficile* infection with clindamycin in critically ill patients. Blood cultures obtained at presentation grew *Pasteurella multocida*, prompting a switch to intravenous Unasyn (ampicillin-sulbactam) at 3 g four times daily. A collateral history from the patient's family revealed the presence of pet cats at home, which the patient later confirmed upon regaining full consciousness.

His clinical condition improved, with significant resolution of sublingual and tongue base edema. He was successfully extubated on day 6 and closely monitored for airway stability post-extubation. As per infectious disease recommendations, Unasyn was switched to ceftriaxone (Rocephin), and the patient completed a 14-day course of antibiotics. Additionally, lisinopril was discontinued at the time of admission to address the possibility of drug-induced angioedema.

At follow-up in the ENT clinic after discharge, the patient remained asymptomatic. A repeat flexible fiberoptic nasopharyngolaryngoscopy revealed a normal upper airway examination without masses or lesions.

## Discussion

Epiglottitis is the inflammation of the epiglottic and supraglottic areas, which can rapidly progress and lead to significant deterioration. The estimated annual incidence of epiglottitis is between 0.97 and 3.1 cases per 100,000 individuals [6], with a mortality rate of 7.1%. Management often necessitates airway intervention, including intubation and cricothyrotomy, requiring a multidisciplinary approach. Infectious epiglottitis can result from bacteremia or direct epithelial invasion [7]. Historically, *Haemophilus influenzae* type B was the leading cause of epiglottitis; however, following widespread immunization, group B *Streptococcus* has become increasingly common. Other implicated pathogens include *Staphylococcus aureus*,* Neisseria meningitidis*, and various viruses and fungi.

A rare bacterial etiology of epiglottitis is *Pasteurella multocida*, a gram-negative rod commonly found in the upper respiratory tracts of animals such as cats, dogs, cattle, rabbits, pigs, and birds. This bacterium can cause both acute and chronic diseases in various animal species. In humans, *Pasteurella multocida* is transmitted primarily through cat (carrier rate: 70%-90%) and dog (carrier rate: 20%-50%) exposure [8]. Most human infections occur via bites, scratches, or oropharyngeal secretions, with immunocompromised individuals particularly more susceptible. While *Pasteurella* infections typically present as localized cellulitis, they can also manifest as meningitis, pneumonia, endocarditis, or bacterial peritonitis, especially in immunocompromised patients or those with liver dysfunction [9].

One of the rare presentations of *Pasteurella multocida* infection is acute epiglottitis. Clinically, *Pasteurella* epiglottitis is indistinguishable from other bacterial causes such as *Haemophilus influenzae* or group B* Streptococcus*, necessitating broad-spectrum antibiotic coverage, including treatment for gram-negative organisms, as well as corticosteroid administration. Among the reported cases, the majority had documented exposure to dogs or cats, with transmission occurring through droplet inhalation or bacteremia following an animal bite or scratch [10]. One case was linked to bird exposure, while another had no identifiable exposure history but had recently traveled to Nigeria before symptom onset.

The most common underlying risk factor associated with *Pasteurella epiglottitis* is tobacco exposure, likely due to impaired mucosal immunity and altered respiratory flora. Of the 14 reported cases, only three patients were immunocompromised, with conditions such as end-stage renal disease (ESRD) and chronic lymphocytic leukemia (CLL) [5,11-13]. Our case also involved a patient with chronic tobacco use (28 pack-year smoking history) and a history of cat scratches. Most cases had favorable outcomes, with only one mortality reported out of the 14 cases [13]. Airway management was required in six cases, including ours, involving intubation, tracheostomy, or cricothyrotomy.

Diagnosis of *Pasteurella* epiglottitis is primarily confirmed via blood culture, although some cases have been confirmed using epiglottic cultures. In our case, *Pasteurella* was identified in two separate blood cultures; however, no sputum cultures were obtained, and no IgA testing was conducted. Antinuclear antibody (ANA) testing was performed to rule out angioedema as a potential cause, yielding negative results.

Once *Pasteurella *is isolated in culture, treatment typically involves penicillin or its derivatives. Amoxicillin combined with a beta-lactamase inhibitor is preferred due to the presence of beta-lactamase production in approximately 16% of infected individuals [10,14]. Alternative treatment options include doxycycline, trimethoprim-sulfamethoxazole, and fluoroquinolones. Anaerobic coverage is sometimes added to address oral flora from the transmitting vector. The duration of treatment generally ranges from five to 14 days. In our case, the patient completed a nine-day course of gram-negative and anaerobic coverage before being discharged on intravenous ceftriaxone via a midline catheter.

As emphasized in the literature, our case highlights the critical importance of early airway management with specialist input to prevent rapid clinical deterioration [15].

## Conclusions

Epiglottitis is a life-threatening condition. *Pasteurella*-associated epiglottitis responds well to appropriate antibiotics. *Pasteurella* is a rare cause of epiglottitis; it requires a high index of suspicion, particularly in those with recent animal contact. Early recognition of symptoms with high clinical suspicion and securing the airway under specialist supervision is vital to prevent rapid deterioration.
